# A longitudinal study on changes in weekend leisure time by age groups in Korea (1999–2019)

**DOI:** 10.1186/s12889-024-18101-z

**Published:** 2024-02-22

**Authors:** Yu-Jin Cha

**Affiliations:** Department of Occupational Therapy, Semyung University, Jecheon, 65 Semyung-ro, Chungbuk 27136 Republic of Korea

**Keywords:** Leisure activity type, Longitudinal study, Time use survey, Weekend

## Abstract

**Background:**

Data analysis was conducted on 20 years (1999–2019) of the Korean Time Use Survey (KTUS) to identify differences and characteristics among each types by extracting latent clusters of weekend leisure activities.

**Methods:**

Using data from the KTUS, we classified weekend leisure time activities into 6 distinct categories. To identify the latent clusters for each year's KTUS data, we utilized latent profile analysis (LPA). Furthermore, independent samples t-tests and one-way ANOVA were conducted to investigate the characteristics of each latent cluster.

**Results:**

As a result of leisure time analysis by survey period, media use accounted for the largest share in all three age groups. The results of the LPA, which included media, sports, culture, and tourism, revealed that the distribution of leisure time for these activities was lower throughout the entire study period.

**Conclusion:**

Based on the results of this study, it is recommended to explore constructive leisure activities and to develop policy measures to promote the domestic leisure industry and leisure consumption.

**Supplementary Information:**

The online version contains supplementary material available at 10.1186/s12889-024-18101-z.

## Background

Leisure activities play an important role in improving the quality of life, individually and socially, as well as in addressing the improvement of the quality of human life, self-realization, and the pursuit of happiness [1]. Leisure is an activity with the special purpose of pursuing pleasure [2]. Satisfaction, rest, and reinvigoration through leisure should improve the overall quality of life and relieve the fatigue and stress generated by working [3]. Leisure as an opportunity for recovery is an important link between work stress and mental and physical health, which sums up the role of leisure in modern society [4].

According to the Korean time use survey (KTUS), except for essential time for maintaining life and meeting social needs (i.e., studying, paid work, unpaid housework, or caring work), the time left over is defined as leisure. The substance of leisure activities includes spending time using media such as reading newspapers and magazines and watching television, pursuing hobbies and entertainment, exercising and playing sports, visiting, engaging in outdoor activities, and simply being idle [5].

The most prominent trends in Korean society over the past decade have been the continuous decrease in working hours and a corresponding increase in leisure time [6]. Socioeconomic changes such as the rise in income levels and the advent of the smart era, the shift from a labor-oriented society to a leisure-oriented society, the expansion of self-oriented values, and the transition from quantitative consumption to a qualitative consumption environment have influenced this change. Leisure activities have also changed steadily based on cultural changes [7]. However, although the average amount of leisure time has increased, it has been observed that the gap between groups in the amount of leisure time spent also increased [8].

According to a recent study on leisure in Korea, the younger the age, the more likely leisure life is centered on travel and friendship [9]. Additionally, middle-aged people with a job prefer relational leisure because they have many social activities [10]. As a result, the younger the age, the stronger the need for self-actualization, so they enjoy more active leisure [11]. Leisure activities change in various ways according to the culture, environment, personal inclinations, lifestyles, and preferences, reflecting the circumstances of the times and sometimes spreading and disappearing like fashion trends [12]. It has been observed that the amount of leisure time varies according to age. In general, leisure time is depicted as a U-shaped curve in which the amount of leisure time is greater in the first half of life and old age while the leisure time available is shorter in adulthood [13]. For this reason, the increasing amount of leisure time after leaving the labor market in old age, together with an aging population, has emerged as an important issue in retirement research [5].

In the case of Korean society, it is necessary to consider when one begins to enjoy leisure time [14]. From 2004 to 2011, the Saturday holiday system was implemented in phases at each business site, and weekends, that is, Saturdays and Sundays, became a time for the general public to enjoy leisure. Introducing a five-day working week means that daily life gains a certain rhythm [5]. Not only the amount of leisure time but also the kind of leisure activities began to be divided according to weekdays and weekends [15]. Institutional changes such as the five-day working week system and five-day study week system have led to changes such as the development of the leisure industry, thus becoming an opportunity to show the possibility that leisure can fully establish itself as a policy target [16].

Analysis of the KTUS indicates that the aspect of leisure that has changed the most over the past decade in Korea has been media use. Internet searches, blog management, and viewing items using a mobile phone such as a phased manufacturing program did not appear in the KTUS of 1999, but 10 years later, in 2009, these elements were added to the large category of leisure activities. In the past, excursions were included in the outdoor activity category, but now, sports club participation is included as an additional category in the detailed classification [5].

In each individual’s use of time, self-reflection by the individual on their role is implied, and the interpretation of time in any situation serves as an effective mechanism for defining oneself and guiding actions. People use the 24 h in the day by allocating the hours as essential time, mandatory time, and leisure time [17]. In other words, the use of time refers to managing time according to one’s situation, and it is essential to plan and use time in a balanced way because it is said to be related to life satisfaction [18]. Time is a significant resource in that choosing what to do at a set time encapsulates the composition of one’s life [19].

Many researchers have classified leisure activity types according to their meaningful criteria [12]. For example, some studies categorize six leisure activities based on participants' motivations: well-being, social, challenge, status, fitness/health and addiction [20]. Meanwhile, other studies classify activities as sports, social, and craft based on the distinctions between 'doing leisure' and 'seeing leisure' [21]. Since the 1990s, various kinds of leisure activities have been classified based on empirical studies, such as cluster analysis and factor analysis, which is different from the conceptual approach [22].

However, existing leisure studies have limitations in capturing the temporality of the weekend in their study designs. In addition, previous studies mainly consisted of a planar analysis in which the total amount of time used was classified based on demographic characteristics through statistics and most of them were the result of cross-sectional studies that observed only the present status based on one time. Therefore, there is a limitation inherent in these studies in that it is difficult to grasp the changes and trends of leisure activities. Consequently, a longitudinal study examining how leisure activities change over time is necessary.

Studying leisure activity patterns provides a wider understanding of how individuals use their free time. This aids in assessing the positive influence of these activities on overall life satisfaction, happiness, and mental health. Analyzing leisure activity patterns also offers insight into the need for recreational services, entertainment, and cultural events, contributing to economic planning and development strategies [23]. Hence, research on leisure patterns is deemed highly significant. However, there has been no attempt to classify types of leisure time use and leisure activity types by time use, nor to understand the characteristics of leisure activities.

This study aimed to identify how time is allocated for those leisure activities engaged in on weekends by type, what types of leisure activities and their content make up weekend leisure, and the changes in the use of leisure time by different age groups over the span of 20 years (1999–2019).

## Methods

### Participants

This research analyzed the data of a 5-year KTUS (1999, 2004, 2009, 2014, and 2019) from nationally approved statistical data published by Statistics Korea. The KTUS was organized by Statistics Korea and was first conducted in 1999 on a 5-year cycle. A total of four surveys examined the changes in leisure time over 20 years. The KTUS used a stratified two-stage cluster sampling method. The target population includes individuals aged 10 and older residing in the Republic of Korea. They are categorized based on the regional stratification index of each city or province. Survey districts were extracted using systematic sampling with probability proportional to size (PPS_SYS). Subsequently, 15 households in each survey district were surveyed, starting from those extracted using simple random extraction (SRS) [24]. According to this data, the time use pattern between rural and urban households was very different on weekdays and weekends by age group; therefore, an urban household group of young people, middle-aged, and older adults was selected to study weekend leisure activities. For the analysis by age group, the subjects were divided into three groups: young peoplㅠe (20–39 years old), middle-aged people (40–64 years old), and older adults (65 years old or older) [25, 26]. The number of respondents was 23,470 in 1999, 18,476 in 2004, 12,215 in 2009, 17,130 in 2014, and 17,228 in 2019, and a total of 88,519 respondents were analyzed.

### Variables and measures

The KTUS is a nationwide time diary that measures the time spent in 24 h on an activity. It replaces the criticism that the existing data on time use reported directly (i.e., self-reported) the time invested in a specific activity which can be affected by perceived social desirability reflecting the subjective intention [19, 27]. It is known that the information on the individual’s time unit behavior provided by the time log guarantees accuracy and objectivity [28]. Such accuracy and objectivity are characteristics of a large-scale survey and national representativeness, as well as strengths of the time use survey. It has been reported that the KTUS is the only essential data that allows a close check on how much time an individual uses for activities in their daily life [29, 30].

The variable for weekend leisure activity was calculated by summing the time used for individual activities included in the six categories by reflecting the classification of the KTUS (S1 Table). For instance, within the category of cultural tourism, the time spent on activities such as movie theater, play·concert, art gallery·museum, watching sports games, and sightseeing·driving activities during the weekend was all added together. This study also used general characteristics such as gender, age, marital status, level of education, monthly income, regular holidays, and weekly working hours. In the KTUS, the lack of time, which was investigated along with the time diary, refers to a state in which an individual perceives that the actual time is insufficient [31]. Using a 4-point Likert scale, the responses to the items ranging from “Always feel busy” (1 point) to “Always feel free” (4 points) were reverse-coded, and the higher the score, the greater the sense of a lack of time. This study was exempted from institutional review board (IRB) deliberation (SMU-EX-2022–07-04), as it fell under public data utilization research.

### Procedure

To examine the changes in the leisure time of the five cohorts in urban groups over 20 years from 1999 to 2019, the weekend leisure activities were classified into six categories by reflecting the classification of the KTUS as it was then (S1 Table). The time for each item was sum scored. In this study, the six categories of weekend leisure activities consisted of cultural tourism, media, sports, gameplay, rest, and others. Among these leisure activities, smoking and doing nothing were included in the rest activity [32].

### Data analysis

This study conducted a descriptive statistical analysis of the general characteristics and time usage of subjects by period using IBM SPSS Statistics 20.0. All other data processing and analyses were performed using R version 4.1.3 statistical software. To identify the latent clusters for each year's KTUS data, latent profile analysis (LPA) was employed, and independent samples t-tests and one-way ANOVA were conducted to examine the characteristics of each cluster based on their respective leisure time activities. The specific data analysis methods are described as follows. Pearson's correlation analysis was performed to examine the relationship between the six categories of leisure time activities in the KTUS data, which was the subject of analysis before conducting the LPA. Second, LPA was employed to investigate how many latent clusters of participants could be identified based on the six categories of leisure time activities in the KTUS data. LPA is a statistical methodology that categorizes individuals into groups, which overcomes the limitation of traditional cluster analysis, which heavily relies on the subjectivity of the analyst [33]. TidyLPA package in R was used for LPA analysis, and the number of latent clusters was determined by considering the Akaike information criteria (AIC) and Bayesian information criteria (BIC) as information indices, the Entropy as an indicator of classification quality, the bootstrap likelihood ratio test (BLRT) as an indicator of statistical significance, and the classification rate. In this case, a better model is indicated by lower values of the information indices and entropy values closer to 1, and significant BLRT results. Finally, to compare and analyze the differences in leisure time according to the latent clusters of the KTUS data, independent two-sample t-tests or one-way ANOVA were conducted. In particular, post-hoc tests for one-way ANOVA were performed using either Tukey's test or Games-Howell's test based on the assumption of equal variances. A Scheffe test was also conducted to analyze the difference in leisure times between the groups by survey period. All tests were conducted considering a significance level of α = 0.05, 0.01, 0.001.

## Results

Table 1 shows the participants’ general characteristics by the survey periods in this study. The gender ratio of men to women was relatively constant at 46.3% for men and 53.7% for women. In terms of age, in 1999, younger adults had the highest participation rate at 49.9%, but in 2019, the middle-aged group recorded the highest rate at 48.9%. Regarding marital status, in 1999, 17.9% were single, 71.7% had a spouse, and 10.4% were widowed. In addition, the divorce rate increased slightly. Regarding education level, in the 1999 survey, 6.5% had no education, 92.4% had a high school degree or lower, and 7.0% had a college degree or higher. In the case of monthly average income, in 1999, those with less than 1 million won showed the highest ratio at 49.5%. In 2019, those with more than 4 million won showed the highest percentage at 35.2%. Those with less than 1 million won accounted for 20.2%, showing changes in the ratio based on the survey period. For working hours per week, in 1999, 0–25 h had the highest rate at 71.8%, and in 2019, 26–50 h had the highest rate at 62.4%, followed by 0–25 h at 15.8%, thus showing changes in the ratio over the survey period.

**Table 1:** General characteristics of the study subjects

	**1999**		**2004**		**2009**		**2014**		**2019**	
	**n**	**(%)**	**n**	**(%)**	**n**	**(%)**	**n**	**(%)**	**n**	**(%)**
Total	23,470		18,476		12,215		17,130		17,228	
Gender										
Male	10,848	(46.2)	8,508	(46.0)	5,752	(47.1)	7,900	(46.1)	7,904	(45.9)
Female	12,622	(53.8)	9,968	(54.0)	6,463	(52.9)	9,230	(53.9)	9,324	(54.1)
Age (years)										
20~39	11,713	(49.9)	7,984	(43.2)	4,690	(38.4)	5,524	(32.2)	4,654	(27.0)
40~64	9,550	(40.7)	8,409	(45.5)	5,830	(47.7)	8,510	(49.7)	8,429	(48.9)
65 over	2,207	(9.4)	2,083	(11.3)	1,695	(13.9)	3,096	(18.1)	4,145	(24.1)
Marital status										
Single	4,191	(17.9)	3,254	(17.6)	1,989	(16.3)	3,017	(17.6)	3,077	(17.9)
Married	16,831	(71.7)	13,192	(71.4)	8,879	(72.7)	11,797	(68.9)	11,576	(67.2)
Education										
Un education	1,536	(6.5)	940	(5.1)	515	(4.2)	609	(3.6)	600	(3.5)
High school or less	21,691	(92.4)	11,311	(61.2)	6,908	(56.6)	8,735	(50.9)	8,620	(50.0)
College degree (<4 years)	243	(1.0)	1,924	(10.4)	1,783	(14.6)	2,813	(16.4)	2,825	(16.4)
College degree (≥4 years)	1,408	(6.0)	3,815	(20.6)	2,584	(21.2)	4,137	(24.2)	4,336	(25.2)
Graduate degree	-	-	486	(2.6)	425	(3.5)	836	(4.9)	847	(4.9)
Monthly income (thousand won)										
<1,000	3,170	(49.5)	11,119	(60.1)	6,235	(51.0)	7,287	(42.5)	1,810	(20.2)
≥1,000 to <2,000	2,338	(36.5)	4,325	(23.4)	3,069	(25.1)	3,976	(23.2)	1,334	(14.9)
≥2,000 to <3,000	645	(10.1)	1,935	(10.5)	1,623	(13.3)	2,843	(16.6)	1,504	(16.8)
≥3,000 to <4,000	181	(2.8)	708	(3.9)	730	(6.0)	1,506	(8.8)	1,163	(13.0)
>4,000	68	(1.1)	389	(2.1)	558	(4.6)	1,518	(8.9)	3,151	(35.2)
Regular holiday										
Sunday	4,432	(41.3)	11,994	(67.6)	2,341	(32.6)	2,740	(27.3)	5,194	(50.9)
Saturday off every other week	735	(6.8)	1,062	(6.0)	697	(9.7)	685	(6.8)	514	(5.0)
2 days a week	760	(7.1)	1,620	(9.1)	2,405	(33.5)	4,586	(45.8)	1,883	(18.4)
Only 1 day every 2 weeks	888	(8.3)	659	(3.7)	324	(4.5)	324	(3.2)	139	(1.4)
Take a break from time to time without a set holiday	3,925	(36.6)	2,415	(13.6)	1,414	(19.7)	1,689	(16.9)	2,482	(24.3)
Others	546	(2.3)	726	(3.9)	576	(4.7)	602	(3.5)	610	(3.5)
Working hours per week (hours)										
0~25	16,851	(71.8)	7,975	(43.2)	698	(9.0)	1,164	(11.0)	1,706	(15.8)
26~50	2,907	(12.4)	5,758	(31.2)	4,159	(53.6)	6,456	(60.8)	6,754	(62.4)
51~75	2,899	(12.4)	4,054	(21.9)	2,440	(31.5)	2,669	(25.1)	2,180	(20.1)
76 over	813	(3.5)	689	(3.7)	457	(5.9)	337	(3.2)	182	(1.7)
The degree to feel lack of time										
Yes, always	5,571	(23.7)	4,338	(23.5)	3,337	(27.3)	3,868	(22.6)	3,558	(20.7)
Yes, sometimes	9,820	(41.8)	7,773	(42.1)	4,885	(40.0)	5,729	(33.4)	5,303	(30.8)
No, almost not	5,527	(23.5)	4,353	(23.6)	2,743	(22.5)	4,969	(29.0)	5,869	(34.1)
No, not at all	2,552	(10.9)	2,012	(10.9)	1,250	(10.2)	2,564	(15.0)	2,498	(14.5)

There was a statistically significant difference in weekend leisure time between the age groups by survey period. In addition, the results of identifying differences by survey period through Scheffe’s test were as shown ins Table 2.

**Table 2:** Time for leisure activities between age groups by survey period. (min)

	**1999(a)**		**2004(b)**		**2009(c)**		**2014(d)**		**2019(e)**		**F**	***P***	**Scheffe (α = 0.05)**
**Category**	*M*	*SD*	*M*	*SD*	*M*	*SD*	*M*	*SD*	*M*	*SD*			
Youth group (20 ~ 39)													
Media	175.32	143.95	156.29	123.8	139.3	112.66	162.61	123.49	169.36	138.29	71.67^***^	0.00	c < b < a
Cultural tourism	5.98	29.42	9.37	34.46	2.11	17.41	4.57	26.37	21.82	57.1	254.20^***^	0.00	c < da < b < e
Sports	13.93	39.66	14.31	37.86	20.45	46.27	21.36	48.28	25.56	51.42	87.00^***^	0.00	ab < cd < e
Games and play	13.64	52.36	30.63	73.47	27.55	68.01	25.15	62.05	32.98	76.19	122.06^***^	0.00	a < d < e
Rest	13.18	26.71	11.39	22.82	16.85	31.96	9.28	18.75	12.61	25.64	62.56^***^	0.00	d < b < a < c
Others (etc.)	12.76	41.52	11.18	38.32	7.59	32.09	10.29	34.35	6.41	27.83	33.44^***^	0.00	ec < d < a
Middle-aged group (40 ~ 64)													
Media	174.13	143.06	175.62	134.78	163.29	120.74	198.48	136.12	174.62	140.8	68.74^***^	0.00	c < aeb < d
Cultural tourism	2.48	21.27	2.95	21.79	1.47	14.1	3.69	23.28	22.63	58.27	635.31^***^	0.00	c < d < e
Sports	23.15	52.95	29.06	58.05	20.26	46	39.00	68.20	26.35	51.24	129.05^***^	0.00	c < a < eb < d
Games and play	12.16	51.75	14.54	47.78	28.39	65.69	8.26	31.69	35.56	81.73	333.83^***^	0.00	d < ab < c < e
Rest	22.69	38.05	18.08	30.78	17.39	33.33	13.60	23.55	12.71	25.06	152.50^***^	0.00	ed < cb < a
Others (etc.)	8.07	32.23	7.16	29.84	6.47	27.55	5.33	23.09	6.00	26.81	12.51^***^	0.00	d < a
Older adults group (65 over)													
Media	241.79	165.93	249.36	157.18	238.28	143.33	275.67	150.60	177.88	140.76	208.95^***^	0.00	e < cab < d
Cultural tourism	4.87	33.81	2.41	18.44	1.71	15.15	1.58	14.88	22.66	56.22	220.72^***^	0.00	d < a < e
Sports	31.92	54.42	41.91	60.24	22.38	48.88	49.53	66.01	26.49	50.79	104.87^***^	0.00	ce < a < b < d
Games and play	21.94	67.6	22.28	58.87	27.84	64.89	13.81	41.23	32.86	77.76	41.45^***^	0.00	d < a < e
Rest	55.46	84.15	41.11	60.04	17.11	34.13	25.23	40.96	14.07	26.77	303.22^***^	0.00	ec < d < b < a
Others (etc.)	2.98	17.27	2.57	18.99	6.52	27.19	2.50	12.93	6.42	27.33	24.79^***^	0.00	dba < ec

^***^p < .001

**Fig. 1:**
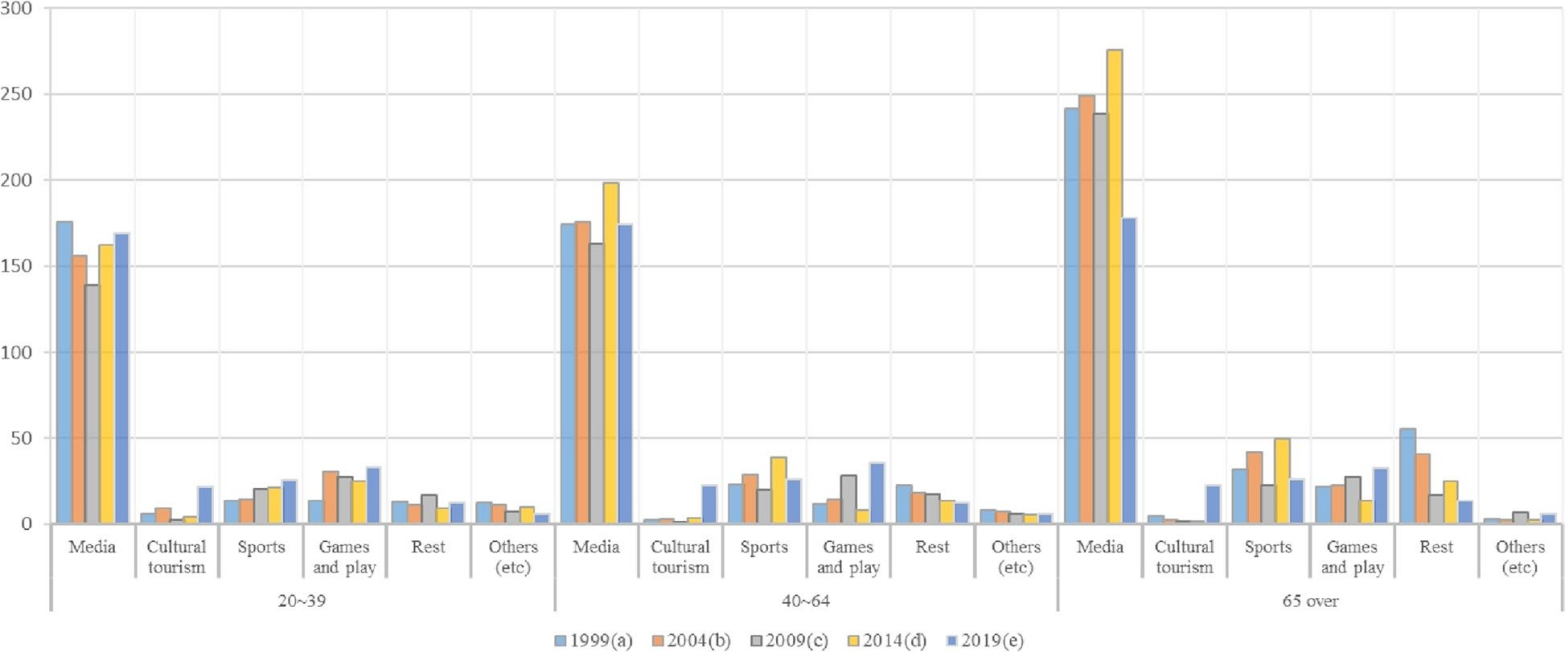
Weekend leisure time between age groups by survey period

Among the young adults group, media use accounted for the largest share in the six types of weekend leisure time analyzed by the survey period. In particular, their media use time in 1999 was the highest at 175.32 min, followed by media use time in 2019 at 169.36 min. Next, games and sports were the most common among the six types of weekend leisure time. For young adults, cultural tourism, sports, and gameplay accounted for the largest share in 2019. For the middle-aged group, media use accounted for the most significant proportion of the six types of weekend leisure time by survey period. In particular, media use time in 2014 was the highest at 198.48 min, while the time in 2009 was the lowest at 163.29 min, with sports and games following. In the case of older adults, media use accounted for the most significant proportion, of which 275.67 min in 2014 was the highest, and 177.88 min in 2019 was the lowest, followed by sports and rest. Comparing the total leisure time between age groups, it was found that older adults had the most leisure time, and media use accounted for the largest share of activity in all three age groups (Fig. 1).

**Fig. 2:**
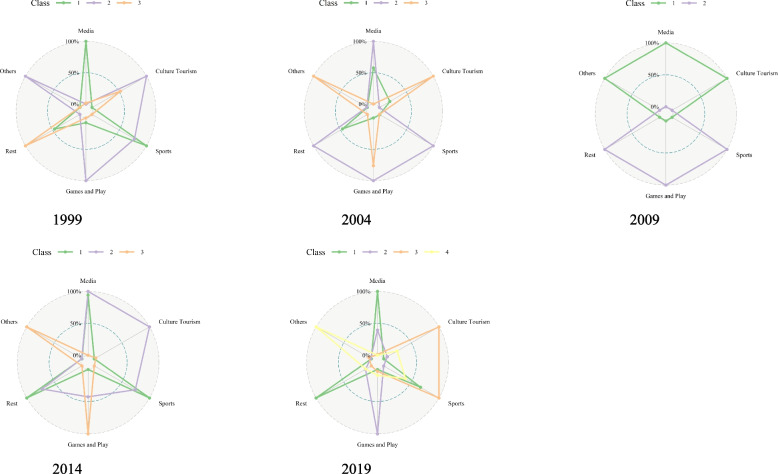
Content of weekend leisure-type activities by survey period

The results of the LPA for determining the number of latent clusters are shown in S2 Table, and the visual representation of leisure time distribution by group based on the results of the LPA is shown in Fig. 2.

A LPA of the 1999 KTUS data identified 3 latent clusters with similar leisure time distributions for culture tourism, sports, games and play, and rest. Class1 was characterized as the 'high media and low others leisure type' due to its high level of 'media' and low level of 'others' compared to other clusters. Class2 was named the 'low media and high others leisure type' because it exhibited a low level of 'media' and high level of 'others' compared to other clusters. Class3 was identified as the 'low media and low others leisure type' due to its low levels of both 'media' and 'others' compared to other clusters.

Based on the 2004 KTUS data, a LPA was conducted and resulted in the determination of 3 latent clusters. Among the 3 leisure time distribution scales, the distribution of culture tourism, games and play, and rest were found to be similar across the three clusters. However, there was an average difference in media leisure time across the three clusters. Class1 was named the 'low sports and low others leisure type' as it showed a low level of 'sports' and 'others' compared to other clusters. Class2 was named the 'high sports and low others leisure type' as it exhibited a high level of 'sports' and a low level of 'others' compared to other clusters. Class3 was named the 'low sports and high others leisure type' as it showed a low level of 'sports' and a high level of 'others' compared to other clusters.

Based on the 2009 KTUS data, 2 latent clusters were identified through LPA. Among the leisure time activity categories, there was a difference in the distribution of 'others' between the 2 clusters. Class 1 showed a higher level of 'others' compared to Class 2 and was named the 'high others leisure type.' Class 2 showed a lower level of 'others' compared to Class 1 and was named the 'low others leisure type.’

Based on the 2014 KTUS data, a LPA was conducted and 3 latent clusters were identified. The distribution of leisure time in the 3 clusters showed similar patterns for sports, games and play, and rest. On the other hand, the media leisure time was found to be similar in Class 1 and Class 2. Class 1 was named ‘low culture tourism and low other leisure type’ because it had a lower level of culture tourism and other leisure activities compared to the other clusters. Class 2 was named ‘high culture tourism and low other leisure type’ because it had a higher level of culture tourism and a lower level of other leisure activities. Class 3 was named ‘low culture tourism and high other leisure type’ because it had a lower level of culture tourism and a higher level of other leisure activities.

**Fig. 3:**
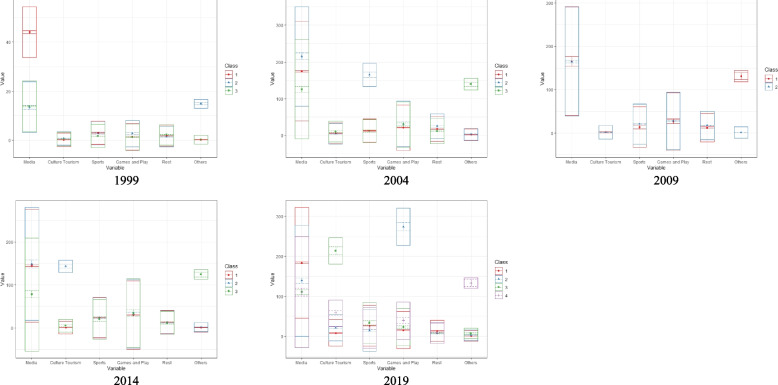
Latent clusters of weekend leisure-type activities by survey period

Based on the 2019 KTUS data, a LPA was conducted and 4 latent clusters were identified. The distribution of leisure time in the four clusters showed similar patterns for sports and rest, while the media leisure time showed differences across all 3 clusters. Class 1 was named ‘low culture tourism, games and play, and other leisure time clusters" because it had lower levels of culture tourism, games and play, and other leisure activities compared to the other clusters. Class 2 was named ‘high games and play and low culture tourism, other leisure time clusters’ because it had a higher level of games and play and lower levels of culture tourism and other leisure activities. Class 3 was named ‘high culture tourism and low games and play, other leisure time clusters’ because it had a higher level of culture tourism and lower levels of games and play and other leisure activities. Class 4 was named ‘high culture tourism and games and play and low other leisure time cluster’ because it had higher levels of culture tourism and games and play and lower levels of other leisure activities (Fig. 3).

The proportion of each latent cluster by survey period is shown in S1 Fig. In 1999, the latent clusters of research participants were differentiated based on media and other leisure time. Other types of leisure activities include personal hobbies, leisurely and the liberal arts study, and entertainment. Class 3 (low media and low other leisure type) accounted for the highest proportion at 82.5%. In 2004, the latent clusters were differentiated based on sports and other leisure time, and Class 1 (low sports and low other leisure type) accounted for the highest proportion at 87.9%. In 2009, the latent clusters were differentiated based on other leisure time, and Class 2 (low other leisure type) accounted for the highest proportion at 95.9%. In 2014, the latent clusters were differentiated based on culture and tourism, and Class 1 (low culture and tourism and low other leisure type) accounted for the highest proportion at 91.5%. In 2019, the latent clusters were differentiated based on culture and tourism, game playing, and other leisure time, and Class 1 (low culture and tourism, game playing, and other leisure time clusters) accounted for the highest proportion at 84.4%.

To examine whether there were differences in leisure time according to classified latent clusters, one-way ANOVA was performed. The results of the ANOVA for the differences between the leisure time categories and the post-hoc test results are presented in (S3 Table). There were statistically significant differences in media, culture and tourism, sports, games and other leisure time categories across latent clusters in 1999, 2004, and 2019. In 2009, there were statistically significant differences in sports, rest, and other leisure time categories across latent clusters. In 2014, there were statistically significant differences in media, culture and tourism, sports, games, and other leisure time categories across latent clusters.

## Discussion

This study focused on leisure activities during weekends in Korea by using the raw data of Statistics Korea’s 5-year cycle (1999–2019) from the KTUS. It also extracted the types of leisure activities to analyze the differences between the types and the characteristics of each type.

As a result of weekend leisure time analysis by survey period, the media accounted for the largest share among the six types of weekend leisure time by survey period for young people, followed by games and sports. For the middle-aged, the media accounted for the most significant proportion, followed by sports and gameplay. As for older adults, media accounted for the largest share. Thus, in conclusion, media use accounted for the largest share in all three age groups.

Media leisure activities such as internet use and social network service management are indeed becoming a trend of the times, and the proportion of them accounting for leisure time is increasing [34]. In terms of spending leisure time between the age groups, there was a high dependence on media in common and a bias toward these activities, with TVs, PCs, and smartphones being used the most [19].

The number one leisure activity that older adults in Korea hope to do in the future is travel. However, since travel is a leisure activity, it is highly likely to be restricted due to time, economics, and physical health. This is why research on the activation of welfare tourism for older adults is vital [35]. It has been shown that if free time becomes a burden after retirement, the time structure of the day collapses and affects well-being negatively [36]. Therefore, it is necessary to present policies related to time management to reflect the needs of older adults in participating in leisure [37].

Although Korea has established various policies for promoting the leisure industry and consumption of leisure, a surprisingly dichotomous phenomenon has been observed in the form of weekend leisure between those who enjoy weekend leisure and those who do not. As a result of the analysis of leisure time between age groups by survey period in this study, the proportion of media and gameplay was high. A recent study on the nature of adolescent leisure, specifically focusing on the routinization of video gaming as a leisure activity, found that playing games for more than 2 h a day was linked to early adolescence, interactions with opposite-sex friends, and parental supervision [38].

In 1999, the latent clusters of research participants were differentiated based on media and other leisure time. Class 3 (low media and low other leisure type) accounted for the highest proportion. In 2004, the latent clusters were differentiated based on sports and other leisure time, and Class 1 (low sports and low other leisure type) accounted for the highest proportion. In 2009, the latent clusters were differentiated based on other leisure time, and Class 2 (low other leisure type) accounted for the highest proportion. In 2014, the latent clusters were differentiated based on culture and tourism, and Class 1 (low culture and tourism and low other leisure type) accounted for the highest proportion. In 2019, the latent clusters were differentiated based on culture and tourism, game playing, and other leisure time, and Class 1 (low culture and tourism, game playing, and other leisure time clusters) accounted for the highest proportion.

In a study analyzing the relationship between leisure types, leisure satisfaction, and happiness among Koreans, it was found that sports-watching activities had the highest level of leisure satisfaction. As people age, it has been observed that preferences and interests in active leisure activities, such as sports, dancing, and travel, tend to decrease. Moreover, there was no significant variation in interest and preference for watching TV, engaging in family-related activities, and visiting friends across different age groups [39]. A study on sports activities and leisure satisfaction among high school students revealed significant differences in the duration of activity participation across leisure satisfaction categories [40]. Engaging in leisure activities through sports was associated with increased life satisfaction and leisure fulfillment among youths. Therefore, promoting participation in sports activities is necessary [41].

Conversely, rest activities showed high levels of leisure dissatisfaction, indicating that participants in those activities experienced lower levels of satisfaction [42]. Despite the increase in leisure time among Korean citizens, it was found that a significant portion of the population primarily engages in rest activities, which makes it challenging for them to enjoy a wide range of leisure pursuits [43]. The findings indicated that leisure activities incorporating physical activity resulted in an enhanced quality of life, greater satisfaction with leisure pursuits, and increased levels of happiness compared to non-physical leisure activities [44, 45]. In the future, it is essential to formulate policies that encourage people to engage in a diverse range of leisure activities rather than merely resting during leisure time. In particular, there is a necessity to promote participation in sports, social events, and other activities rather than resting.

Based on the results of LPA, which examined the distribution of weekend leisure time by period, including media, sports, culture and tourism, and other leisure activities, it was observed that these activities were performed less frequently throughout the entire study period (2009 ~ 2019). These findings highlight the need for various policy measures to promote the domestic leisure industry and encourage leisure consumption.

The limitations of this study are that, due to the nature of the data, the analysis focused on the amount of time available per day. Therefore, it was not feasible to distinguish leisure activities occurring on a weekly or annual basis. Additionally, since secondary data from Statistics Korea were used, there were limitations in the selection and operational definition of variables. It is particularly regrettable that related characteristics could not be analyzed because the survey did not include any psychological or social variables that could have been used as mediating factors. Although lifetime data had sufficient data on time, the question about the subjective time experience felt on weekdays was measured as a single question. Lastly, the most recent survey for KTUS in Korea was conducted four years ago in 2019, so there is a limitation in that it did not include research on changes in leisure activity time due to the COVID-19 pandemic. A follow-up study should examine how this will change in the future by using a time-use survey questionnaire that corrects and accounts for these limitations. Despite these limitations, this study is significant in that it categorized weekend leisure time usage and types by period and age group using representative data from a nationwide time use survey.

## Conclusion

This study aimed to focus on leisure activities during the weekend in Korea and identify the differences and characteristics of each type by extracting the latent clusters of leisure activities. The results showed the following: (1) Through the analysis of leisure time across survey periods, regardless of age group, media use emerged as the predominant activity, occupying the largest share of leisure activities for all three age groups. (2) The proportion of each latent cluster by survey period. In 1999, Class 3 (low media and low other leisure type) had the highest proportion, followed by Class 1 (low sports and low other leisure type) in 2004, Class 2 (low other leisure type) in 2009, and Class 1 (low culture and tourism and low other leisure type) dominating in 2014, with Class 1 (low culture and tourism, game playing, and other leisure time clusters) accounting for the highest proportion in 2019. (3) Based on the results of LPA, which examined the distribution of weekend leisure time by period, including media, sports, culture and tourism, and other leisure activities, it was observed that these activities were performed less frequently throughout the entire study period (2009 ~ 2019). Therefore, it is essential to design education and programs featuring leisure activities to enhance the quality of life on both national and social levels. Based on the results, it has been confirmed that there is a need for various policy measures to promote the domestic leisure industry and encourage leisure consumption.

### Supplementary Information

**Supplementary file 1.**

**Supplementary file 2.**

**Supplementary file 3.**

**Supplementary file 4.**

## Data Availability

Publicly available datasets have been used for this study. The data used to support the findings of this study were provided by Korean National Statistical Office (KNSO) under license: Statistics Korea (http://kostat.go.kr/portal/eng/).

## Abbreviations

AIC: Akaike information criteria
BIC: Bayesian information criteria
BLRT: Bootstrap likelihood ratio test
KTUS: Korean time use survey
LPA: Latent profile analysis
