# Supplementary figures and images for: A longitudinal study on changes in weekend leisure time by age groups in Korea (1999–2019)
Source: BMC Public Health. 2024 Feb 22;24:552. doi: 10.1186/s12889-024-18101-z (PMC10882758; doi:10.1186/s12889-024-18101-z)

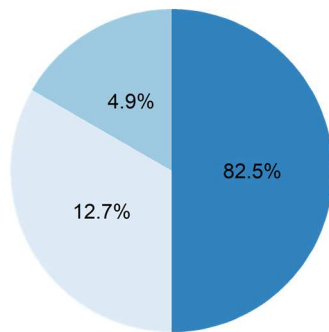

1999

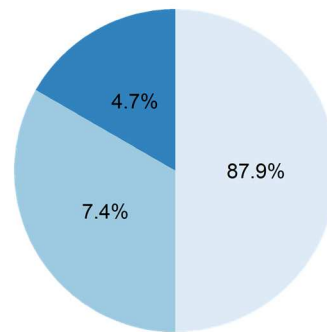

2004

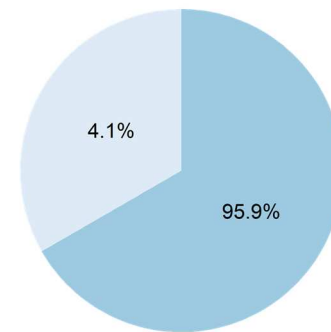

2009

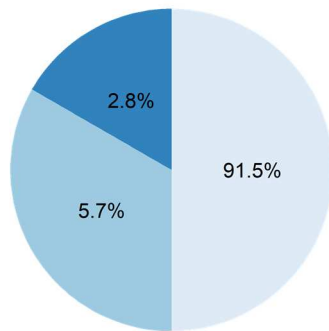

2014

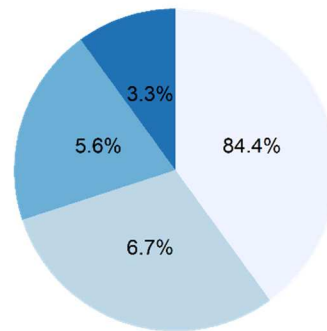

2019

S1 Fig. Proportion of potential cluster by survey periods.

Supplement: Supplementary file 1 [file 12889_2024_18101_MOESM1_ESM.pdf]
