# Supplementary material for: A longitudinal study on changes in weekend leisure time by age groups in Korea (1999–2019)
Source: BMC Public Health. 2024 Feb 22;24:552. doi: 10.1186/s12889-024-18101-z (PMC10882758; doi:10.1186/s12889-024-18101-z)
Supplement: Supplementary file 2 [file 12889_2024_18101_MOESM2_ESM.docx]

| **Leisure types** | **Sub - types** |
| --- | --- |
| Media | Reading books |
|  | Reading newspaper |
|  | Reading magazine |
|  | Watching TV shows |
|  | Watching video |
|  | Listening to radio |
|  | Listening to audio |
|  | Searching the internet |
|  | Others (etc) |
| Cultural tourism | Movie theater |
|  | Play∙concert |
|  | Art gallery∙museum |
|  | Watching sports games |
|  | Sightseeing∙driving |
|  | Others (etc) |
| Sports | Walking∙stroll |
|  | Running∙jogging |
|  | Climbing |
|  | Cycling∙inline skating |
|  | Individual workout |
|  | Ball game sports |
|  | Fishing∙hunting |
|  | Others (etc) |
| Game and play | Group play∙games |
|  | Computer/PC games |
|  | Mobile games |
|  | Others (etc) |
| Rest | Do nothing and rest |
|  | Smoking |
| Others (etc) | Personal hobbies |
|  | Leisure∙liberal arts study |
|  | Entertainment |
|  | Others (etc) |

**S1 Table** Types of leisure time activities.
