# Supplementary material for: A longitudinal study on changes in weekend leisure time by age groups in Korea (1999–2019)
Source: BMC Public Health. 2024 Feb 22;24:552. doi: 10.1186/s12889-024-18101-z (PMC10882758; doi:10.1186/s12889-024-18101-z)
Supplement: Supplementary file 3 [file 12889_2024_18101_MOESM3_ESM.docx]

The results of the LPA for determining the number of latent clusters according to the weekend leisure time classification of KTUS in 1999 are as shown in [S2-1], and as a result of the comprehensive analysis, a model with 3 latent groups was identified as the optimal choice.

**S2-1** Results of the LPA for determining the number of latent clusters (1999) (N=23,470)

| Classes | AIC | BIC | Entropy | BLRT  p-value | Latent Class distribution rate (%) | | | | | |
| --- | --- | --- | --- | --- | --- | --- | --- | --- | --- | --- |
|  |  |  |  |  | **1** | **2** | **3** | **4** | **5** | **6** |
| 2 | 852,151.22 | 852,304.42 | 0.21 | $0.07$ | 0.0 | 100 |  |  |  |  |
| 3 | 823,386.52 | 823,596.17 | 0.89 | ${0.01}^{*}$ | 12.7 | 4.9 | 82.5 |  |  |  |
| 4 | 837,032.90 | 837,299.00 | 0.51 | ${0.01}^{*}$ | 74.0 | 8.4 | 14.3 | 3.4 |  |  |
| 5 | 827,086.68 | 827,409.22 | 0.35 | ${0.01}^{*}$ | 94.9 | 0.1 | 0.0 | 5.0 | 0.0 |  |
| 6 | 812,553.08 | 812,932.07 | 0.46 | ${0.01}^{*}$ | 5.0 | 3.3 | 0.0 | 3.4 | 14.5 | 73.8 |

$p<{0.05}^{*}$The optimal model choice from the latent clusters model is highlighted in the row.

The results of the LPA for determining the number of latent clusters according to the weekend leisure time classification of KTUS in 2004 are as shown in [S2-2], and as a result of the comprehensive analysis, a model with 3 latent groups was identified as the optimal choice.

**S2-2** Results of the LPA for determining the number of latent clusters (2004) (N=18,476)

| Classes | AIC | BIC | Entropy | BLRT  p-value | Latent Class distribution rate (%) | | | | | |
| --- | --- | --- | --- | --- | --- | --- | --- | --- | --- | --- |
|  |  |  |  |  | **1** | **2** | **3** | **4** | **5** | **6** |
| 2 | 1,173,822.82 | 1,173,971.48 | 0.77 | ${0.01}^{*}$ | 15.9 | 84.1 |  |  |  |  |
| 3 | 1,146,395.04 | 1,146,598.47 | 0.97 | ${0.01}^{*}$ | 87.9 | 7.4 | 4.7 |  |  |  |
| 4 | 1,144,272.35 | 1,144,530.55 | 0.88 | ${0.01}^{*}$ | 6.7 | 76.4 | 12.2 | 4.7 |  |  |
| 5 | 1,141,030.74 | 1,141,343.71 | 0.61 | ${0.01}^{*}$ | 5.3 | 13.7 | 76.4 | 4.7 | 0.0 |  |
| 6 | 1,116,786.57 | 1,117,154.31 | 0.73 | $1.00$ | 75.4 | 3.5 | 0.0 | 4.4 | 11.8 | 4.9 |

$p<{0.05}^{*}$ The optimal model choice from the latent clusters model is highlighted in the row.

The results of the LPA for determining the number of latent clusters according to the weekend leisure time classification of KTUS in 2009 are as shown in [S2-3], and as a result of the comprehensive analysis, a model with 2 latent groups was identified as the optimal choice.

**S2-3** Results of the LPA for determining the number of latent clusters (2009) (N=12,215)

| Classes | AIC | BIC | Entropy | BLRT  p-value | Latent Class distribution rate (%) | | | | | |
| --- | --- | --- | --- | --- | --- | --- | --- | --- | --- | --- |
|  |  |  |  |  | **1** | **2** | **3** | **4** | **5** | **6** |
| 2 | 742,272.94 | 742,413.73 | 1.00 | $1.00$ | 4.1 | 95.9 |  |  |  |  |
| 3 | 742,294.62 | 742,487.29 | 0.59 | $1.00$ | 95.8 | 0.0 | 4.2 |  |  |  |
| 4 | 742,300.63 | 742,545.18 | 0.46 | $0.36$ | 95.7 | 4.3 | 0.0 | 0.0 |  |  |
| 5 | 741,057.35 | 741,353.76 | 0.50 | ${0.01}^{*}$ | 0.0 | 0.0 | 4.1 | 80.2 | 15.7 |  |
| 6 | 741,061.61 | 741,409.90 | 0.45 | $1.00$ | 0.0 | 4.1 | 78.7 | 0.0 | 14.9 | 2.3 |

$p<{0.05}^{*}$ The optimal model choice from the latent clusters model is highlighted in the row.

The results of the LPA for determining the number of latent clusters according to the weekend leisure time classification of KTUS in 2014 are as shown in [S2-4], and as a result of the comprehensive analysis, a model with 3 latent groups was identified as the optimal choice.

**S2-4** Results of the LPA for determining the number of latent clusters (2014) (N=17,130)

| Classes | AIC | BIC | Entropy | BLRT  p-value | Latent Class distribution rate (%) | | | | | |
| --- | --- | --- | --- | --- | --- | --- | --- | --- | --- | --- |
|  |  |  |  |  | **1** | **2** | **3** | **4** | **5** | **6** |
| 2 | 1,079,559.26 | 1,079,706.48 | 0.83 | ${0.01}^{*}$ | 84.6 | 15.4 |  |  |  |  |
| 3 | 1,038,296.06 | 1,038,497.52 | 1.00 | ${0.01}^{*}$ | 91.5 | 5.7 | 2.8 |  |  |  |
| 4 | 1,046,227.50 | 1,046,483.20 | 0.91 | ${0.01}^{*}$ | 78.1 | 5.9 | 13.2 | 2.8 |  |  |
| 5 | 1,027,576.56 | 1,027,886.50 | 0.74 | $1.00$ | 86.1 | 5.7 | 0.0 | 2.8 | 5.5 |  |
| 6 | 1,035,293.02 | 1,035,657.20 | 0.58 | $1.00$ | 78.0 | 13.5 | 0.0 | 0.0 | 2.8 | 5.7 |

$p<{0.05}^{*}$ The optimal model choice from the latent clusters model is highlighted in the row.

The results of the LPA for determining the number of latent clusters according to the weekend leisure time classification of KTUS in 2019 are as shown in [S2-5], and as a result of the comprehensive analysis, a model with 4 latent groups was identified as the optimal choice.

**S2-5** Results of the LPA for determining the number of latent clusters (2019) (N=17,228)

| Classes | AIC | BIC | Entropy | BLRT  p-value | Latent Class distribution rate (%) | | | | | |
| --- | --- | --- | --- | --- | --- | --- | --- | --- | --- | --- |
|  |  |  |  |  | **1** | **2** | **3** | **4** | **5** | **6** |
| 2 | 1,115,181.63 | 1,115,328.96 | 0.24 | ${0.01}^{*}$ | 100 | 0.0 |  |  |  |  |
| 3 | 1,083,756.90 | 1,083,958.51 | 0.98 | ${0.01}^{*}$ | 3.7 | 89.3 | 7.0 |  |  |  |
| 4 | 1,072,028.95 | 1,072,284.84 | 0.98 | ${0.01}^{*}$ | 84.4 | 6.7 | 5.6 | 3.3 |  |  |
| 5 | 1,088,371.56 | 1,088,681.73 | 0.68 | ${0.01}^{*}$ | 5.9 | 0.0 | 14.0 | 72.8 | 7.3 |  |
| 6 | 1,087,418.35 | 1,087,782.80 | 0.65 | ${0.01}^{*}$ | 0.0 | 69.6 | 5.7 | 4.6 | 7.8 | 12.2 |

$p<{0.05}^{*}$ The optimal model choice from the latent clusters model is highlighted in the row.
