# Supplementary material for: A longitudinal study on changes in weekend leisure time by age groups in Korea (1999–2019)
Source: BMC Public Health. 2024 Feb 22;24:552. doi: 10.1186/s12889-024-18101-z (PMC10882758; doi:10.1186/s12889-024-18101-z)
Supplement: Supplementary file 4 [file 12889_2024_18101_MOESM4_ESM.docx]

**S3-1.** Results of the ANOVA for the differences between the leisure time categories and the post-hoc test results (1999) (N=23,470)

| Sub variable | Class | n | Mean | SD | F | Games-Howell |
| --- | --- | --- | --- | --- | --- | --- |
| Media | 1  2  3 | 2,970  1,144  19,356 | 46.75  13.46  13.99 | 10.35  12.75  9.60 | ${13205}^{***}$ | ${1>2, 3}^{**}$ |
| Culture Tourism | 1  2  3 | 2,970  1,144  19,356 | 0.16  0.75  0.47 | 1.33  3.41  2.80 | ${54.74}^{***}$ | ${2>1}^{**}, 3^{*}$ ${3>1}^{**}$ |
| Sports | 1  2  3 | 2,970  1,144  19,356 | 3.13  2.82  1.70 | 5.60  6.16  4.45 | ${102.23}^{***}$ | ${1, 2>3}^{**}$ |
| Games and Play | 1  2  3 | 2,970  1,144  19,356 | 1.41  2.78  1.29 | 4.85  7.21  5.32 | ${23.83}^{***}$ | ${2>1, 3}^{**}$ |
| Rest | 1  2  3 | 2,970  1,144  19,356 | 1.81  1.48  2.18 | 3.80  2.85  4.30 | ${38.299}^{***}$ | ${3>1, 2}^{**}$  ${1>2}^{*}$ |
| Others | 1  2  3 | 2,970  1,144  19,356 | 0.22  15.02  0.28 | 1.06  6.07  1.20 | ${3375.9}^{***}$ | ${2>1, 3}^{**}$  ${3>1}^{*}$ |

$$p<{0.05}^{*}, p<{0.01}^{**}, p<{0.001}^{***}$$

**S3-2.** Results of the ANOVA for the differences between the leisure time categories and the post-hoc test results (2004) (N=18,476)

| Sub variable | Class | n | Mean | SD | F | Games-Howell |
| --- | --- | --- | --- | --- | --- | --- |
| Media | 1  2  3 | 16,248  1,364  864 | 175.22  211.39  125.80 | 135.88  136.89  114.94 | ${127.27}^{***}$ | ${2>1, 3}^{**}$  ${1>3}^{**}$ |
| Culture Tourism | 1  2  3 | 16,248  1,364  864 | 5.52  4.35  10.39 | 27.78  23.44  35.09 | ${10.028}^{***}$ | ${3>1, 2}^{**}$ |
| Sports | 1  2  3 | 16,248  1,364  864 | 12.67  168.61  11.66 | 25.91  67.96  34.40 | ${3549.7}^{***}$ | ${2>1, 3}^{**}$ |
| Games and Play | 1  2  3 | 16,248  1,364  864 | 21.20  31.90  29.35 | 60.86  71.39  61.33 | ${20.659}^{***}$ | ${2, 3>1}^{**}$ |
| Rest | 1  2  3 | 16,248  1,364  864 | 17.54  24.84  11.27 | 33.37  42.92  21.15 | ${56.333}^{***}$ | ${2>1, 3}^{**}$  ${1>3}^{*}$ |
| Others | 1  2  3 | 2,970  1,144  19,356 | 1.85  3.14  139.34 | 9.47  13.85  57.30 | ${2486}^{***}$ | ${3>1, 2}^{**}$  ${2>1}^{*}$ |

$$p<{0.05}^{*}, p<{0.01}^{**}, p<{0.001}^{***}$$

**S3-3.** Results of the ANOVA for the differences between the leisure time categories and the post-hoc test results (2009) (N=12,215)

| Sub variable | Class | n | Mean | SD | t |
| --- | --- | --- | --- | --- | --- |
| Media | 1  2 | 498  11,717 | 168.49  164.32 | 124.78  125.23 | $0.7294$ |
| Culture Tourism | 1  2 | 498  11,717 | 1.91  1.74 | 13.27  15.69 | $0.2298$ |
| Sports | 1  2 | 498  11,717 | 13.47  20.93 | 40.99  46.71 | ${-3.9517}^{***}$ |
| Games and Play | 1  2 | 498  11,717 | 26.47  28.06 | 58.37  66.80 | $-0.5236$ |
| Rest | 1  2 | 498  11,717 | 12.23  17.35 | 21.97  33.29 | ${-4.969}^{***}$ |
| Others | 1  2 | 498  11,717 | 133.51  1.53 | 51.5  8.6 | ${57.163}^{***}$ |

$$p<{0.05}^{*}, p<{0.01}^{**}, p<{0.001}^{***}$$

**S3-4.** Results of the ANOVA for the differences between the leisure time categories and the post-hoc test results (2014) (N=17,130)

| Sub variable | Class | n | Mean | SD | F | Games-Howell/Tukey |
| --- | --- | --- | --- | --- | --- | --- |
| Media | 1  2  3 | 15,677  979  474 | 144.40  148.47  77.67 | 132.74  130.38  90.49 | ${122.84}^{***}$ | ${1, 2> 3}^{**}$ |
| Culture Tourism | 1  2  3 | 15,677  979  474 | 0.78  143.02  4.81 | 6.48  52.38  23.74 | ${3609.5}^{***}$ | ${1>2, 3}^{**}$  ${2>3}^{**}$ |
| Sports | 1  2  3 | 15,677  979  474 | 23.90  22.89  19.96 | 46.88  43.44  47.07 | $1.8102$ |  |
| Games and Play | 1  2  3 | 15,677  979  474 | 29.32  31.43  34.26 | 79.78  76.93  76.02 | $1.1727$ |  |
| Rest | 1  2  3 | 15,677  979  474 | 13.18  12.58  11.03 | 26.84  23.06  25.46 | $1.6847$ |  |
| Others | 1  2  3 | 15,677  979  474 | 1.02  1.62  123.86 | 6.62  10.92  51.31 | ${1357.8}^{***}$ | ${3>1, 2}^{**}$ |

$$p<{0.05}^{*}, p<{0.01}^{**}, p<{0.001}^{***}$$

**S3-5.** Results of the ANOVA for the differences between the leisure time categories and the post-hoc test results (2019) (N=17,228)

| Sub variable | Class | n | Mean | SD | F | Games-Howell |
| --- | --- | --- | --- | --- | --- | --- |
| Media | 1  2  3  4 | 14,535  1,160  957  576 | 183.41  139.19  111.26  110.42 | 143.39  110.68  98.60  112.33 | ${238.05}^{***}$ | ${1>2, 3, 4}^{**}$  ${2>3, 4}^{**}$ |
| Culture Tourism | 1  2  3  4 | 14,535  1,160  957  576 | 8.47  21.79  213.44  58.39 | 21.29  34.26  95.25  55.53 | ${1669.6}^{***}$ | ${3>1, 2, 4}^{**}$  ${4>1, 2}^{**}$  ${2>1}^{**}$ |
| Sports | 1  2  3  4 | 14,535  1,160  957  576 | 26.83  14.47  33.01  21.79 | 50.57  36.37  71.50  47.85 | ${43.375}^{***}$ | ${3>1, 2, 4}^{*}$  ${1, 4>2}^{**}$ |
| Games and Play | 1  2  3  4 | 14,535  1,160  957  576 | 15.58  274.35  23.03  39.36 | 35.89  102.97  47.71  73.90 | ${2435.4}^{***}$ | ${2>1, 3, 4}^{**}$  ${4>1, 3}^{**}$  ${3>1}^{**}$ |
| Rest | 1  2  3  4 | 14,535  1,160  957  576 | 13.88  8.55  7.92  8.51 | 26.75  17.64  17.22  19.11 | ${62.377}^{***}$ | ${1>2, 3, 4}^{**}$ |
| Others | 1  2  3  4 | 14,535  1,160  957  576 | 1.36  2.97  7.26  133.37 | 7.90  12.88  18.59  53.70 | ${1193.2}^{***}$ | ${4>1, 2, 3}^{**}$  ${3>1, 2}^{**}$  ${2>1}^{**}$ |

$$p<{0.05}^{*}, p<{0.01}^{**}, p<{0.001}^{***}$$
